# Isolation and Characterization of a Novel Myophage Abp9 Against Pandrug Resistant *Acinetobacater baumannii*

**DOI:** 10.3389/fmicb.2020.506068

**Published:** 2020-09-08

**Authors:** Lingli Jiang, Jingjie Tan, Yi Hao, Qi Wang, Xiaorui Yan, Dali Wang, Li Tuo, Zairong Wei, Guangtao Huang

**Affiliations:** ^1^Department of Burn and Plastic, Affiliated Hospital of Zunyi Medical University, Zunyi, China; ^2^Life Sciences Institute, Zunyi Medical University, Zunyi, China

**Keywords:** *Acinetobacter baumannii*, phage therapy, biofilm, pandrug resistance, cell lysis

## Abstract

*Acinetobacter baumannii (A. baumannii)* has emerged as one of the most troublesome pathogens in health care institutions. *A. baumannii* can cause a wide range of diseases in humans, including pneumonia and septicemia. Phage therapy has drawn great interest from medical researchers as a potential way to control infections by antibiotic-resistant *A. baumannii*. Using a pandrug-resistant clinical *A. baumannii* isolate AB_ZY_9 as an indicator, we isolated a lytic phage Abp9 from hospital sewage. Abp9 belongs to *myoviridae* family and shows a wider host range of 12%. Abp9 contains a linear double-stranded DNA genome of 44,820 bp with a G + C content of 37.69%. The Abp9 genome contains 80 open reading frames, but lacks any known virulence genes or lysogen-formation genes. In a systemic *A. baumannii* infection mouse models, Abp9 treatment showed good therapeutic effects. We have also observed an excellent lytic activity against *A. baumannii* in biofilm form of growth *in vitro*. All of these suggest that Abp9 is a good candidate for the phage therapy against drug-resistant *A. baumannii* infections.

## Introduction

*A. baumannii* is an aerobic bacillus of non-fermentative, Gram-negative bacteria. *A. baumannii* usually inhabits in soil, sewage, surface of medical appliances and hospital environments, from where it can cause bacteremia, pneumonia, cephalomeningitis and infections of urinary tract, skin and soft tissues, especially under immunosuppressive status (Maragakis and Perl, 2008; Peleg et al., 2008; Freire et al., 2016; Gong et al., 2016). *A. baumannii* is one of the leading pathogens causing hospital infection (Hauck et al., 2012; Bahador et al., 2015; Gong et al., 2016; Huang et al., 2016). In recent years, increasing attention was paid to *A. baumannii* as its ability to quickly develop antibiotic resistance. This adaption is mainly attributed though its plastic genome, which rapidly mutates when facing adversity and stress. *A. baumannii* could also form robust biofilm with in the wound and on occlusive dressings, as well as on most abiotic surfaces. These characteristics make it difficult to eliminated *A. baumannii* completely. World Health Organization has declared *A. baumannii* as one of the most serious ESKAPE organisms (Pereira et al., 2018). Meanwhile, the Centers for Disease Control and Prevention (CDC) categorized multidrug resistance (MDR) *A. baumannii* as a serious threat.

Phage therapy is a promising therapeutic method that could be deployed against the global superbug crisis. PhagoBurn was the first randomized controlled trial to investigate phage therapy which was launched in June 2013 and ended in February 2017 (Jault et al., 2019). Over the past few years, a growing number of researchers have focused on isolating and validating the use of phages for therapy and prophylaxis in the war against drug-resistant *A. baumannii*. Currently, 21 *A. baumannii* phages have been sequenced and released in databases. AP22, was the first reported *A. baumannii* phage of *myoviridae* family (Popova et al., 2012). A detailed genome bioinformatic analysis of the *A. baumannii* phage AB1 was also reported in 2012 (Li et al., 2012). The therapeutic efficacy of phage therapy against carbapenem-resistant *A. baumannii* (CRAB) infections in Galleria mellonella larvae and a mouse model of acute pneumonia had been reported (Jeon et al., 2019). In our previous study, phage Abp1 could rescue mice from Pandrug Resistant *A. baumannii* infections (Yin et al., 2017). Meanwhile, on-demand phage isolation has also showed promising results (Mattila et al., 2015). Even more, personalized bacteriophage-based therapeutic cocktails was also used in necrotizing pancreatitis caused by MDR *A. baumannii* (Schooley et al., 2017). Combination of bacteriophages and antibiotics had also been reported to save a patient’s leg with trauma-related left tibial *A. baumannii* infection (Nir-Paz et al., 2019). So far, none clinical trial on *A. baumannii* phages is reported (PubMed and web of science) or registered^1^.

We recently isolated *A. baumannii* strain AB_ZY_9 from the femoral vein catheter of a patient. AB_ZY_9 is sensitive to polymyxin and resistant to aminoglycosides, carbapenems, cephalosporins, tetracyclines, and quinolones. Along with its pandrug- resistant phenotype, ABZY9 can also form biofilms in plates. We then sought to isolate a lytic bacteriophage for ABZY9 from hospital sewage. The present study reports on the successful isolation of a lytic phage we have called Abp9, which has a wide host range. After biological characterization and genome dissection, we found that Abp9 effectively cleared ABZY9-produced biofilms, and reduced the mortality of rats infected with this bacterium.

## Materials and Methods

### Bacterial Strains

*A. baumannii* strain AB_ZY_9 was isolated from a burn patient. After identified by VITEK, AB_ZY_-9 was further confirmed as *A. baumannii* species by MLST analysis and 16S rRNA sequencing (27f:5′-AGAGTTTGATCCTGGCTCAG-3′, 1492r:5′ -GGTTACCTTGTTACGACT T-3′) (Huang et al., 2013). For MLST analysis, seven conserved genes (*gltA, gyrB, gdhB, recA, cpn60, gpi, and rpoD*) were sequenced (Huang et al., 2016).

### Bacteriophage Isolation and Transmission Electron Microscopy (TEM)

Abp9 was isolated from sewage using AB_ZY_9 as the host bacterium with previous method (Huang et al., 2013). Briefly, a total of 1 L filtered hospital sewage was incubated overnight with 100 mL AB_ZY_9 (OD_600_ around 1.0). The supernatant was collected and tested for phages with a double-layer plate. The plaques can be observed after incubation at 37°C overnight. The phage particles were prepared by CsCl gradient ultracentrifugation (Nasukawa et al., 2017). CsCl-purified Abp9, at a concentration of 10^11^ PFU/mL, was deposited onto carbon-coated copper grids and allowed to adsorb for 15 min. The phage particles were negatively stained using 2% (w/v) potassium phosphotungstate and visualized with a transmission electron microscope. The length and width of Abp9 was measured with ImageJ. Phage DNA was isolated using a phage genome kit (Norgen, Canada).

### One-Step Growth Assay

For one-step growth experiments, a previous described method was used. Briefly, after phage adsorption at room temperature for a 15 min, centrifugation at 13,000 g for 30 s, followed by removing the supernatant. The pelleted cells were resuspended in 5 mL of preheated (37°C) LB broth and incubated at 37°C. Samples were taken at 10 min intervals. Phage titers were immediately obtained. Experiments were repeated at least three times with duplicate samples.

### Host-Range Determination

For host-range test, a total of 97 (AB_ZY_9 not included) clinical *A. baumannii* isolates were tested with a previous described method. These 97 clinical isolates were collected in our previous study (Huang et al., 2016). These clinical isolates belong to 10 different STs and most of them (94/97) are MDR *A. baumannii* isolates. Briefly, a total of 200 μL of *A. baumannii* cells (OD_600_ around 1.0) was mixed with 3 mL of melted 0.6% agar (50°C), and this mixture was poured onto 1.5% solid agar to make double-layer agar plates. After solidification for 10 min, 5 μL of 10^8^ PFU/mL of Abp9 phages was spotted onto the double-layered agar plates. After 12 h incubation, we observe whether any lysis plaques had emerged.

### Genome Sequencing

Sequencing libraries Were generated using the TruSeq DNA Sample Preparation Kit (Illumina, United States) and the Template Prep Kit (Pacific Biosciences, United States). Genome sequencing Was performed by the Personal Biotechnology Company (Shanghai, China) on the Pacific Biosciences platform and the Illumina Miseq platform. Data assembly Was conducted using SPAdes (Bankevich et al., 2012) and A5-miseq (Coil et al., 2015) to construct the scaffolds and contigs. Canu software (Koren et al., 2017) was used to assemble the data obtained From PacBio platform sequencing. Subsequently, all assembled results Were integrated to generate a complete sequence.

### Bioinformatic Analysis

Open reading frames (ORFs) were predicted using GeneMark (version 4.32)^2^. The functions of the proteins encoded by each ORF were predicted using the BLASTp database^3^. Gene prediction was performed by Glimmer 3.02 (Delcher et al., 1999). tRNAscan-SE (Lowe and Eddy, 1997), RNAmmer (Lagesen et al., 2007), and Rfam (Burge et al., 2013) were used to find transfer RNA, ribosomal RNA and other non-coding RNAs, respectively. The whole genome was submitted to https://www.ncbi.nlm.nih.gov/genbank/ (NCBI accession number: MN166083). For the phylogenetic analysis, a phylogenetic tree was constructed with the Molecular Evolutionary Genetic Analysis (MEGA version 7.0) package based on a comparison of whole genome sequences.

### Thermal and pH Stability of Abp9

To test the thermal stability of Abp9, a 1.5 mL volume of phages (5 × 1010 142 PFU/mL) was incubated for 15 min at 20, 30, 40, 50, 60, and 70°C, after which 100 μL aliquots were withdrawn to calculate the phage concentrations. After 10-fold dilution, 10 μL of each diluted sample was separately combined with 200 μL of host bacteria, the solutions incubated for 15 min, mixed with 3 mL of 0.75% LB agar, and then poured onto solid-agar plate to determine the phage titers. For the pH stability assay, aliquots were collected 30 min after incubation in liquid media ranging from pH 3.0 to 12.0.

### Phage Therapy in a Systemic Infection Model

A total of 36 nine-week-old SD mouse (20–30 g) were randomly divided into 3 groups. The mouse were anesthetized with continuous inhalation of isoflurane. 200 μL of PBS containing 5.0 × 10^7^ CFU AB_ZY_9 cells was injected intraperitoneally into the mice. Immediately after infection, phage (5.0 × 10^8^ PFU) in PBS were injected intraperitoneally on the same side of the mouse used for the bacterial injection. Phage were injected again after 12 h. The animals were monitored for 7 days and the survival rate of each group was calculated.

### Biofilm Removal Ability of Abp9

*A. baumannii* biofilm formation assays were conducted as described previously (O’Toole and Kolter, 1998). Briefly, 100 μL of an overnight *A. baumannii* culture was 5 mL of fresh LB medium. After 2–3 h, the cells were harvested when the OD600 reached 1.0 (around 5 × 108 CFU/mL). After diluting the medium 50 times, 100 μL of culture (around 1 × 107 CFU/mL) was added to each well of a 96-well plate (Beaver, China), and the plate was incubated at 37°C for 24 h. A 10 μL aliquot (1 × 108 PFU) of phage lysate was added to each well in the phage treatment group. In the control group, the same volume of TM buffer (10 mM Tris⋅HCl, pH 8.0/10 mM MgSO4) was added. Non-attached bacteria were removed by tipping out and rinsing the wells three to four times by immersing the plates into a tub of water, then pouring the water out of the wells. After formaldehyde fixation, crystal violet was used for biofilm staining. The stained dye was washed out with 95% ethanol and subjected to OD595 measurement using a micro plate reader.

### Ethics Statement

All animal experiments were complied with the International Guiding Principles for Biomedical Research involving Animals (1985) and were approved by the Laboratory Animal Welfare and Ethics Committee of Zunyi Medical University. The approval number for the animal experiment is 2019-2-032.

### Statistical Analysis

Data were analyzed by one-way analysis of variance (ANOVA) or log-rank test (Mantel-Cox) as appropriate. A value of *P* < 0.05 was considered significant.

## Results

### Abp9 Is a Lytic Myophage Phage

**FIGURE 1:**
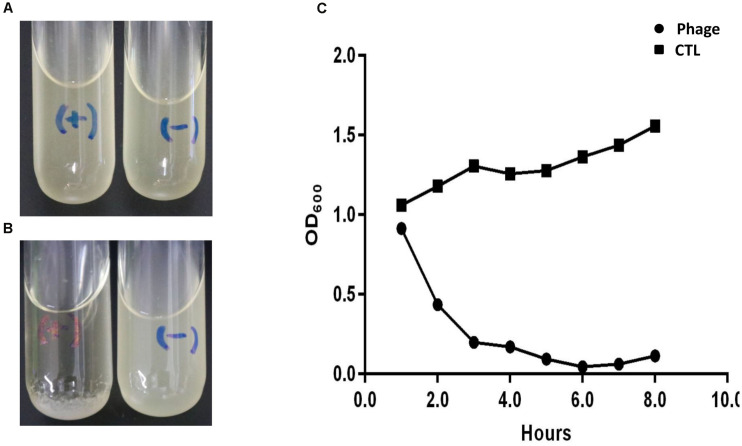
Lytic result of Abp9 in LB liquid medium. **(A,B)** AB_ZY_-9 culture in the logarithmic growth phase become clear again after adding Abp9 (marked with) for 3 h. Meanwhile, AB_ZY_-9 culture without Abp9 become more turbid. “+” means adding phage Abp9, “–” means adding same volume of LB medium. **(C)** Trends of absorbance value (OD_600_) in each group after adding Abp9.

When the OD600 of AB_ZY_9 culture reached to 1.0, a total of 10 μL (5 × 10^7^ PFU) Abp9 were added into the culture. Three hours later, the AB_ZY_9 culture become almost clear and bacterial residue settles to the bottom (Figures 1A,B). As shown in Figure 1C, OD_600_ of AB_ZY_9dropped quickly in phage group. The OD600 decreased to almost zero 6 h after adding phage. Meanwhile, the viable bacterial counts of *A. bauma*nnii ABZY9 decreased from 5.4 × 108 CFU/mL to 4.5 × 106 CFU/mL.

**FIGURE 2:**
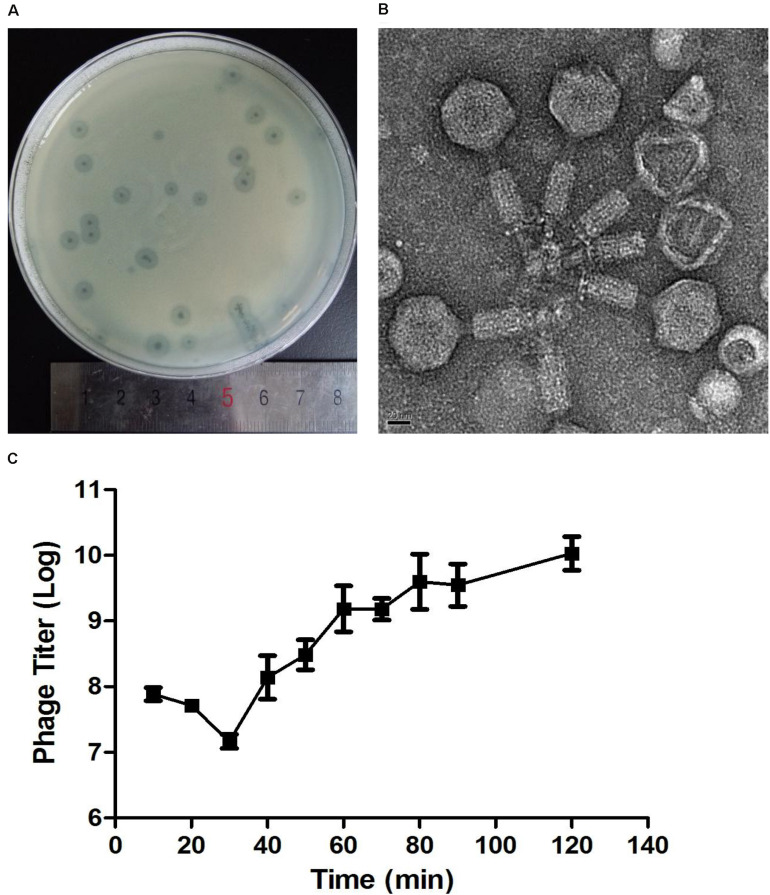
Plaques, TEM and One-step growth curves for Abp9. **(A)** Abp9 formed clear lytic plaques on double-layered plates, the diameters of which measured 1–3 mm. **(B)** TEM analysis of Abp9. Abp9 has an icosahedral head and a contractable tail. **(C)** Abp9 has a latent period of 30–40 min. Data represent the mean ± SD from three replicates. Burst size is the number of phages produced per-infected cell and equal to 158 PFU/cell.

After incubation for 8 h at 37°C, Abp9 formed clear plaques on the double-layered plate (Figure 2A). The plaques were clear with diameters of around 1–3 mm, and semi-transparent halos were apparent on the host’s bacterial lawns (Figure 2A). As Figure 2B shows, Abp9 is a *Myoviridae* family member. Abp9 has an icosahedral head (as seen by the simultaneous presence of hexagonal and pentagonal capsids) of 55.3 ± 2.2 nm in diameter. The head is separated from the tail sheath by a 16.4 ± 4.1 nm long neck. Abp9 possess a contractible tail measuring (101.7 ± 4.0) × (17.9 ± 1.2) nm. As shown in Figure 2C, after a latent 30 min phase, Abp9 burst out of the cells 40 min after their infection. The burst size of the phage was approximately158 PFU/cell.

### Phage Stability

**FIGURE 3:**
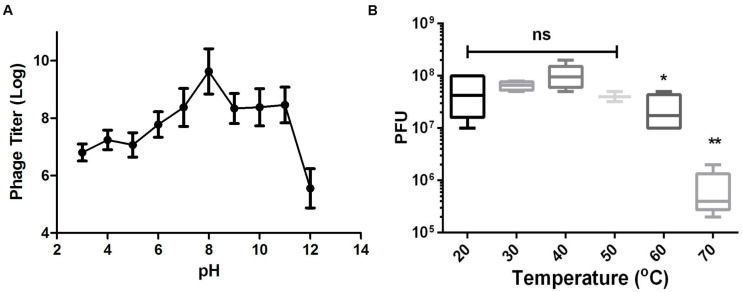
Thermal and pH stability of Abp9. **(A)** pH stability. Phage Abp9 was incubated at the indicated pH conditions for 30 min. Data were obtained from three independent experiments and are shown as mean ± standard deviation. **(B)** Thermal stability of Abp9. Phage titers start to decrease when the temperature over 50 degree. Data are presented as mean ± standard deviations from three replicates. “ns” means no significant difference, “*” means *p* < 0.05, “**” means *p* < 0.01.

As shown in Figure 3A, Abp9 also exhibits good pH stability. Abp9 survival was high from pH 7.0 to 11.0. Abp9 titers decreased significantly between pH 3.0 and 6.0, and from 11.0 to 12.0. Abp9 is also relatively thermal-stable at 50°>C (Figure 3B). No difference of phage titers were detected at 20, 30, 40, and 50°>C. However, phage titers decreased around 10-folds after incubation at 60°>C for 15 min. No more than 1% of phage were viable after 15 min incubation at 70°C. These results demonstrate that Abp9 retains activity well above 37°>C, which simplifies its storage and delivery. The broad range of thermal and pH stability facilitates the application of Abp9 in phage therapy.

### General Features of the Abp9 Genome

The entire Abp9 genome was sequenced. After quality control, 5,208,458 reads and 27,585 genome coverage were obtained. The final genome assembly yielded a 44,820 bp linear, dsDNA molecule with 37.69% G + C content. A total of 80 ORFs larger than 100 bp were identified using ORF Finder. ORF density is around 1.8 genes per kb. The average length of a gene was approximately 487 bp.

**FIGURE 4:**
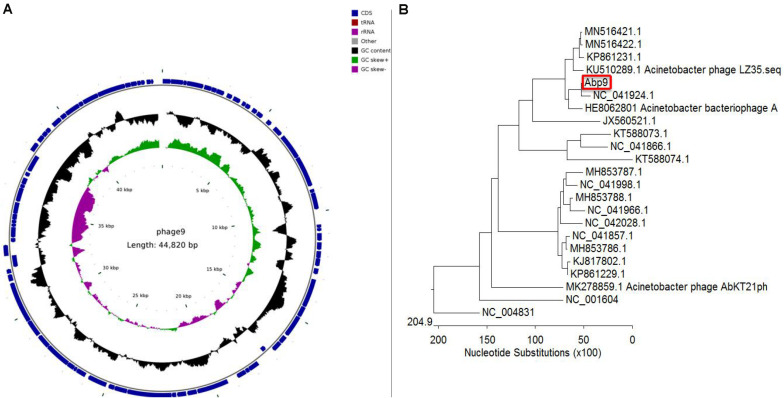
Circular genome map and genome comparison of phage Abp9. **(A)** The innermost circle represents GC skew (purple: GC skew–; green: GC skew+); the black circle in the middle represents the GC content (black outwards indicate GC content greater than 37.69%, and black inwards indicate the opposite); the outermost circle shows 80 predicted ORFs. **(B)** The phylogenetic tree was constructed with MEGA using the whole genome sequences with MEGA. A total of 21 sequenced *A. baumannii* phage genomes, together with SP6 and T7 phage, were aligned together with the Abp9.

As shown in Supplementary Table 1, genome annotation of a Abp9 was listed. Sixty seven genes were encoded on the positive strand and 13 genes on the negative strand. Of these 80 gens, only 16 genes were function-known after bioinformatic analysis. Five structural proteins were identified, including head protein, base plate protein and tail protein. DNA helicase, endodeoxyribonuclease, and two terminase subunits were identified. Some regulatory genes were also identified in Abp9, including antirepressor protein, superinfection immunity protein, transcriptional dual regulator, secretion activator protein and superinfection immunity protein. Figure 4A shows the genome map of Abp9. The genome sequence was deposited in the NCBI genome database under GenBank accession number MN166083.

### Genome-Wide Comparison

A total of 21 sequenced *A. baumannii* phage genomes were downloaded and compared with Abp9. Most of these 21 genomes fall into two major groups. As shown in Figure 4B, Abp9 shares the highest similarity with *Acinetobacter* phageWCHABP12 (NC_041924.1), a Chinese isolate, followed by A. bacteriophage AP22 (HE806280.1), a Russian isolate. The query coverage forWCHABP12 and AP22 was 85 and 64%, respectively. In contrast, Abp9 was found to share least similarity with *Acinetobacter* phage phiAC-1 (JX560521.1).

### Host Range and Phage Therapy in a Systemic Infection Model

**FIGURE 5:**
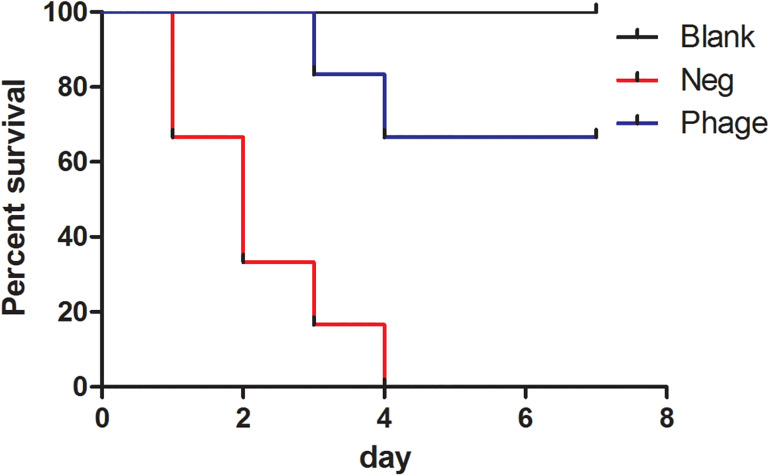
Survival rate of Abp9 therapy. Red line indicates negative group, blue line indicates phage group and black line indicates blank group.

We subjected 97 clinical isolates of *A. baumannii* clinic strains and found 12 of them were sensitive to Abp9. Only one of the 12 phage-sensitive clinic isolates are antibiotic-sensitive strain and others are MDR strains. The 12 strains also belonged to 5 different STs (ST368 6 isolates, ST208 2 isolates, ST191 1 isolates, ST136 1 isolates, new STs 2 isolates). No shared antibiotic resistance phenotype and molecule ST types among these 12 phage-sensitive strains were identified. In addition, *A. baumannii* clinic strain ATCC19606, *Pseudomonas aeruginosa* strain PAO1 and Escherichia coli strains JM109, DH5a were also tested and all showed resistance to the Abp9. To further investigate the potential therapeutic effects of Abp9 against systemic infections, we devised an *A. baumannii* mouse infection model. As shown in Figure 5, infected mouse in the negative group succumbed gradually to the infection. In the no phage treatment group, the infected mouse started to die on the first day post-infection, with all 12 of them dying after 1 week. In the phage treatment group, the infected mouse started to die at 3 days post-infection and eight mouse survived after 1 week, suggesting that Abp9 therapy has potential clinical utility for the control of systemic infections.

### *In vitro* Biofilm Lytic Activity of Abp9

**FIGURE 6:**
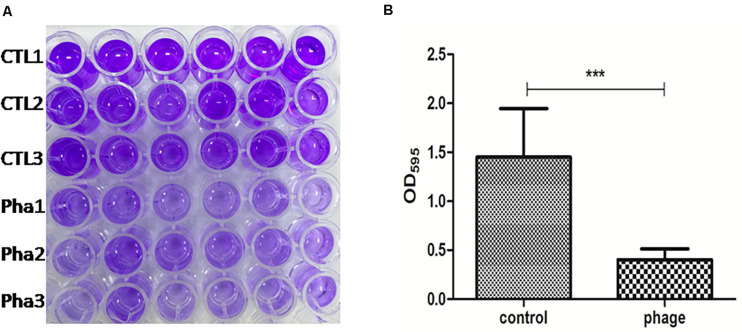
Biofilm degradation Abp9 phage. **(A)** CTL1, CTL2, and CTL3 represent three control groups and Pha1, Pha2, and Pha3 represent three phage groups. **(B)** Biofilm density decreased significantly in the phage group measured by OD_595_. Data are presented as mean ± SD from six replicates. *** indicates a significant difference.

Infections involving biofilm formation by the *A. baumannii* are very difficult to eliminate. To test the biofilm removal ability of Abp9, a biofilm model *in vitro* was used in this study. As shown in Figure 6, the biofilm density tested by OD_595_ decreased significantly from 1.80 to 0.50 after co-cultured with phage Abp9 for 2 h. This result indicates that Abp9 could clear biofilm efficiently.

## Discussion

Due to the narrow host range of phages, on-demand phage isolation of specific pathogens might be a compromise strategy for phage therapy. In this study, a lytic phage was isolated from hospital sewage with the host bacteria AB_ZY_9, which was isolated from the catheter of a burn patient. The morphology observed by electron microscope indicated that Abp9 belongs to *myoviridae* (Figure 2B).

Previously, several *A. baumannii* myophages had been reported (Lee et al., 2011; Jin et al., 2012; Essoh et al., 2019; Oliveira et al., 2019; Popova et al., 2019), including Abp53, vB_AbaM_B9, ZZ1, Aci01-1, Aci02-2, Aci05, and AM24. Abp53 has a DNA genome of 95 kb, which encodes several proteins similar to those encoded by host *A. baumannii* and *Klebsiella* phage phiKO2 reported by Lee et al. (2011). vB_AbaM_B9 has a genome of 93,641 bp and encodes 167 predicted proteins, with a G + C content of 33.6%. vB_AbaM_B9 could specifically lytic from without in strains of the K45 and K30 capsule types (Oliveira et al., 2019). AM24 also contains 167 ORFs, with 127 function-unknown genes. However, it has a linear double-stranded DNA genome of 97,177 bp, with a G + C content of 37.3% (Popova et al., 2019). ZZ1 possesses a double-stranded DNA with a total length of 166 kb. Bioinformatic analysis of the phage whole genome sequence further suggested that ZZ1 was more likely to be a new member of the *Myoviridae* phages (Jin et al., 2012). The Aci01-1, Aci02-2, and Aci05 genomes were 103, 104, and 102 kb long with direct terminal repeats (DTRs) of 1,184, 1,198, and 1,151 bp, respectively. The genome size and architecture and the presence of a DTR and tRNAs suggest that these phages may be members of the Felixounavirinae subfamily (Essoh et al., 2019) Abp9 possess a relatively small genome than the other three myophages of *A. baumannii*. Abp9 contains a linear double-stranded DNA genome of 44,820 bp with 37.7% G + C content. BLASTP search predicted the functions of 16 ORFs, most of which resembled proteins of *Acinetobacter* phage WCHABP1 and *Acinetobacter* phage WCHABP12.

Abp9 shows good thermal stability from 20 to 50°>C, and phage titers decreased significantly at temperatures over 60°>C (Figure 3B). Thus, this level of thermal stability may facilitate the application of Abp9 in phage therapy. In rats with severe burn wound infection model, Abp9 local administration treatment showed a higher survival rate than the negative group (Supplementary Figure S1). However, there is no significant difference (*p* = 0.096). Based on our systemic mice infection model, Abp9 shows potential for use in controlling *A. baumannii* infections, which is important because this bacterium can form biofilms on the surfaces of catheters or wounds. Phage therapy is being explored as a new tool form clearing biofilms. Here, we found that Abp9 was able to remove biofilms formed by ABZY9 (Figure 6). The depolymerase activities of some phages, which degrade components of the biofilm exopolymeric matrix, enable them to infect the inner cells of biofilms (Carson et al., 2010). Thus, it is possible that the depolymerases produced by phages used in clinic as biofilm-penetrating antibiotics (Verma et al., 2010).

In the present study, we have isolated a lytic phage against a clinical *A. baumannii* strain AB_ZY_-9 *A. baumannii* strain, which was isolated from the femoral vein catheter of a patient. Our TEM analysis indicates that Abp9 belongs to the *Myoviridae* bacteriophage family. Abp9 exhibits a broad range of thermal stability and can clear biofilms efficiently *in vitro*. Thus, Abp9 has potential for treating patients suffering *A. baumannii* infections.

## Data Availability Statement

The datasets generated for this study can be found in the NCBI (accession number MN166083).

## Ethics Statement

The animal study was reviewed and approved by the Laboratory Animal Welfare and Ethics Committee of Zunyi Medical University.

## Author Contributions

GH and ZW conceived and designed this study. LJ, JT, YH, and QW carried out the experiments. XY, LT, and LJ analyzed the data. DW and LJ drafted the manuscript. All authors have read and approved the final manuscript.

## Conflict of Interest

The authors declare that the research was conducted in the absence of any commercial or financial relationships that could be construed as a potential conflict of interest.

## Footnotes

1 https://clinicaltrials.gov/

2 http://topaz.gatech.edu/GeneMark/

3 https://blast.ncbi.nlm.nih.gov/Blast.cgi?PAGE=Proteins

## Supplementary Material

FIGURE S1 Survival rate of Abp9 therapy in severe burn infection rat model.
