# Corrigendum: Isolation and Characterization of a Novel Myophage Abp9 Against Pandrug Resistant *Acinetobacater baumannii*

**DOI:** 10.3389/fmicb.2020.625283

**Published:** 2020-12-03

**Authors:** Lingli Jiang, Jingjie Tan, Yi Hao, Qi Wang, Xiaorui Yan, Dali Wang, Li Tuo, Zairong Wei, Guangtao Huang

**Affiliations:** ^1^Department of Burn and Plastic, Affiliated Hospital of Zunyi Medical University, Zunyi, China; ^2^Life Sciences Institute, Zunyi Medical University, Zunyi, China

**Keywords:** *Acinetobacter baumannii*, phage therapy, biofilm, pandrug resistance, cell lysis

In the published article, there was an error in affiliations **1** and **2**. Instead of “**1 Department of Burn and Plastic, The First Affiliated Hospital of Zunyi Medical University, Zunyi, China, 2 Department of Microbiology, Zunyi Medical University, Zunyi, China**,” it should be “**1 Department of Burn and Plastic, Affiliated Hospital of Zunyi Medical University, Zunyi, China; 2 Life Sciences Institute, Zunyi Medical University, Zunyi, China**.”

The authors apologize for this error and state that this does not change the scientific conclusions of the article in any way. The original article has been updated.

