# Supplementary figures and images for: Isolation and Characterization of a Novel Myophage Abp9 Against Pandrug Resistant Acinetobacater baumannii
Source: Front Microbiol. 2020 Sep 8;11:506068. doi: 10.3389/fmicb.2020.506068 (PMC7506109; doi:10.3389/fmicb.2020.506068)

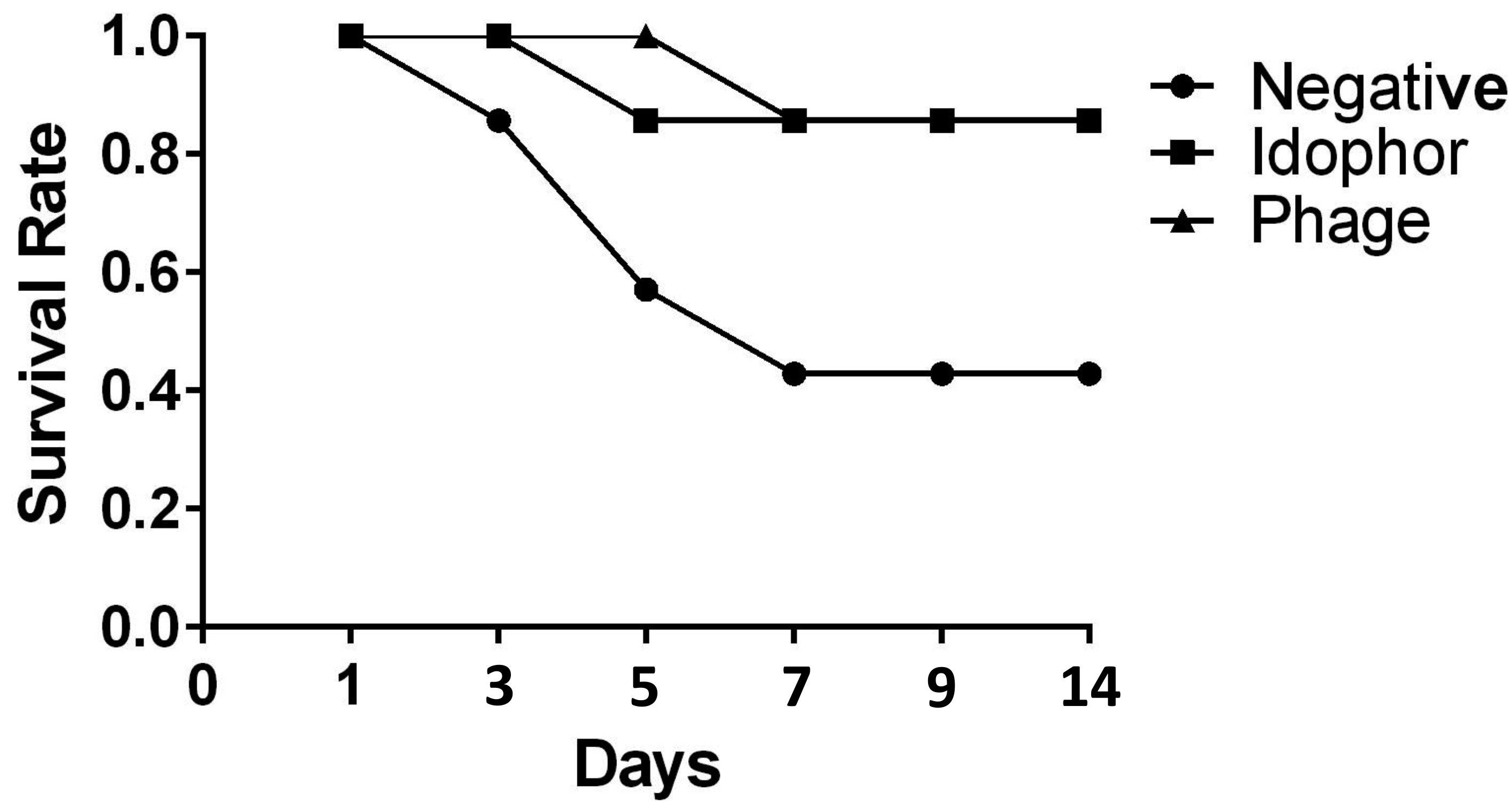

Supplement: FIGURE S1 — Survival rate of Abp9 therapy in severe burn infection rat model. [file Image_1.JPEG]
