# Supplementary material for: Isolation and Characterization of a Novel Myophage Abp9 Against Pandrug Resistant Acinetobacater baumannii
Source: Front Microbiol. 2020 Sep 8;11:506068. doi: 10.3389/fmicb.2020.506068 (PMC7506109; doi:10.3389/fmicb.2020.506068)
Supplement: Supplementary file 2 [file Table_1.DOCX]

Supplementary Table 1. Annotation of Abp1 genome

| **Name** | **Length(aa)** | **Strand** | **Description** |
| --- | --- | --- | --- |
| **phage9_1** | **126** | **+** | **hypothetical protein** |
| **phage9_2** | **191** | **+** | **hypothetical protein** |
| **phage9_3** | **54** | **+** | **hypothetical protein** |
| **phage9_4** | **255** | **+** | **hypothetical protein** |
| **phage9_5** | **117** | **+** | **hypothetical protein** |
| **phage9_6** | **82** | **+** | **hypothetical protein** |
| **phage9_7** | **120** | **+** | **crossover junction endodeoxyribonuclease** |
| **phage9_8** | **86** | **+** | **hypothetical protein** |
| **phage9_9** | **115** | **+** | **hypothetical protein** |
| **phage9_10** | **61** | **+** | **hypothetical protein** |
| **phage9_11** | **77** | **+** | **global DNA-binding transcriptional dual regulator** |
| **phage9_12** | **80** | **+** | **hypothetical protein** |
| **phage9_13** | **97** | **+** | **hypothetical protein** |
| **phage9_14** | **78** | **+** | **hypothetical protein** |
| **phage9_15** | **143** | **+** | **terminase small subunit** |
| **phage9_16** | **393** | **+** | **terminase large subunit** |
| **phage9_17** | **475** | **+** | **putative portal protein** |
| **phage9_18** | **57** | **+** | **hypothetical protein** |
| **phage9_19** | **256** | **+** | **hypothetical protein** |
| **phage9_20** | **54** | **+** | **hypothetical protein** |
| **phage9_21** | **48** | **+** | **hypothetical protein** |
| **phage9_22** | **117** | **+** | **hypothetical protein** |
| **phage9_23** | **111** | **+** | **hypothetical protein** |
| **phage9_24** | **120** | **+** | **hypothetical protein** |
| **phage9_25** | **91** | **+** | **hypothetical protein** |
| **phage9_26** | **442** | **+** | **hypothetical protein** |
| **phage9_27** | **164** | **+** | **hypothetical protein** |
| **phage9_28** | **335** | **+** | **hypothetical protein** |
| **phage9_29** | **117** | **+** | **hypothetical protein** |
| **phage9_30** | **150** | **+** | **hypothetical protein** |
| **phage9_31** | **74** | **-** | **hypothetical protein** |
| **phage9_32** | **168** | **+** | **hypothetical protein** |
| **phage9_33** | **62** | **+** | **hypothetical protein** |
| **phage9_34** | **145** | **+** | **hypothetical protein** |
| **phage9_35** | **487** | **+** | **hypothetical protein** |
| **phage9_36** | **147** | **+** | **hypothetical protein** |
| **phage9_37** | **141** | **+** | **hypothetical protein** |
| **phage9_38** | **70** | **+** | **hypothetical protein** |
| **phage9_39** | **676** | **+** | **lysozyme-like domain protein** |
| **phage9_40** | **198** | **+** | **hypothetical protein** |
| **phage9_41** | **92** | **+** | **hypothetical protein** |
| **phage9_42** | **296** | **+** | **hypothetical protein** |
| **phage9_43** | **156** | **+** | **hypothetical protein** |
| **phage9_44** | **48** | **+** | **hypothetical protein** |
| **phage9_45** | **117** | **+** | **hypothetical protein** |
| **phage9_46** | **394** | **+** | **baseplate J-like protein** |
| **phage9_47** | **208** | **+** | **hypothetical protein** |
| **phage9_48** | **258** | **+** | **putative tail fiber protein** |
| **phage9_49** | **634** | **+** | **hypothetical protein** |
| **phage9_50** | **106** | **+** | **hypothetical protein** |
| **phage9_51** | **70** | **+** | **hypothetical protein** |
| **phage9_52** | **189** | **-** | **NUMOD4 motif protein** |
| **phage9_53** | **170** | **+** | **secretion activator protein** |
| **phage9_54** | **68** | **-** | **hypothetical protein** |
| **phage9_55** | **168** | **-** | **hypothetical protein** |
| **phage9_56** | **53** | **-** | **hypothetical protein** |
| **phage9_57** | **60** | **-** | **hypothetical protein** |
| **phage9_58** | **97** | **-** | **hypothetical protein** |
| **phage9_59** | **188** | **-** | **hypothetical protein** |
| **phage9_60** | **226** | **-** | **ERF superfamily protein** |
| **phage9_61** | **60** | **-** | **hypothetical protein** |
| **phage9_62** | **110** | **-** | **hypothetical protein** |
| **phage9_63** | **67** | **-** | **hypothetical protein** |
| **phage9_64** | **272** | **-** | **hypothetical protein** |
| **phage9_65** | **141** | **+** | **hypothetical protein** |
| **phage9_66** | **108** | **+** | **hypothetical protein** |
| **phage9_67** | **80** | **+** | **hypothetical protein** |
| **phage9_68** | **71** | **+** | **hypothetical protein** |
| **phage9_69** | **158** | **+** | **hypothetical protein** |
| **phage9_70** | **96** | **+** | **hypothetical protein** |
| **phage9_71** | **237** | **+** | **antirepressor protein KilAC domain protein** |
| **phage9_72** | **70** | **+** | **hypothetical protein** |
| **phage9_73** | **251** | **+** | **hypothetical protein** |
| **phage9_74** | **433** | **+** | **replicative DNA helicase** |
| **phage9_75** | **88** | **+** | **hypothetical protein** |
| **phage9_76** | **95** | **+** | **hypothetical protein** |
| **phage9_77** | **60** | **+** | **hypothetical protein** |
| **phage9_78** | **58** | **+** | **hypothetical protein** |
| **phage9_79** | **70** | **+** | **putative superinfection immunity protein** |
| **phage9_80** | **37** | **+** | **hypothetical protein** |
